# Democratizing computational skills: evaluating an asynchronous microlearning framework for cloud-based data analytics in health services research

**DOI:** 10.3389/fpubh.2026.1868973

**Published:** 2026-06-16

**Authors:** Yulia A. Levites Strekalova, Rachel Liu-Galvin, Mishal Khan, Eva Lee, Ernest Alema-Mensah, Elizabeth Ofili

**Affiliations:** 1 Department of Health Services Research, Management, and Policy, College of Public Health and Health Professions, University of Florida, Gainesville, FL, United States; 2 Data and Analytics Innovation Institute, Atlanta, GA, United States; 3 Morehouse School of Medicine, Atlanta, GA, United States

**Keywords:** asynchronous learning, computational skills, data analytics, health service research, workforce development

## Abstract

**Background:**

Public health is undergoing a digital transformation, with increasing reliance on data-driven decision-making that requires proficiency in computational tools. However, traditional curricula often emphasize theoretical knowledge over applied technical skills, contributing to gaps in workforce readiness. This study evaluated a pilot remote, asynchronous microlearning course designed to expand access to digital skills–specifically R and Google Colab–among students from historically underrepresented backgrounds within the Research Centers in Minority Institutions (RCMI) network.

**Methods:**

A three-week course, “*Introduction to Cloud Data Analytics for Health Services Research*,” was delivered via a Learning Management System. Students (*N* = 19) completed three modules: (1) R and RStudio fundamentals, (2) cloud-based analysis of data from the Health Information for National Trends Survey 7 using Google Colab, and (3) scientific abstract writing. Evaluation included a pre- and post-program five-item objective knowledge assessment, retrospective self-rated competencies (1–5 scale), and a post-program satisfaction survey.

**Results:**

Mean objective knowledge scores increased significantly from 3.58 to 4.37 (*p* = .039). Participants reported statistically significant improvements in eight of nine self-rated competencies (*p* < .05), with the largest gains in installing R/RStudio and navigating the interface. Satisfaction was high across domains, particularly for “value for academic development” (*M*= 4.5/5.0).

**Conclusion:**

Brief, asynchronous microlearning experiences can effectively build foundational computational skills and expand access to training for students in low-resourced settings. While technical competencies can be developed within short, flexible formats, more complex skills such as scientific communication may require additional instructional time and support.

## Introduction

Health services and public health practice are undergoing a digital transformation that is reshaping how professionals address population health challenges (1). The shift toward data-driven decision-making has moved the field away from intuition-based approaches toward evidence-based strategies that leverage large datasets, predictive analytics, and real-time surveillance systems to guide policy and practice (2). Computational tools have become increasingly central to health services research, enabling complex analyses that were previously impractical or impossible (3–5). As a result, there is a growing need for digitally literate public health professionals who can effectively use tools such as R, RStudio, and cloud-based platforms to analyze and communicate health data (6, 7). As public health agencies increasingly rely on advanced analytics to track epidemics, assess health equity, and evaluate interventions, integrating digital skills into public health education is essential to preparing the next generation of practitioners to address evolving health challenges (5, 6, 8).

Contemporary public health is therefore experiencing a broader transition toward a model defined by evidence-based, data-driven decision-making. This evolution is fueled by the expanding availability of large-scale datasets, advances in predictive analytics, and the growing need for real-time disease surveillance in an interconnected world (9). However, this rapid pace of technological change has outpaced the pedagogical evolution of many academic institutions (10). A widening gap persists between traditional public health curricula, which often emphasize theoretical knowledge, and the technical competencies now required in practice, including advanced data manipulation and cloud-based computing (9, 11). Addressing this gap is critical, as the ability to leverage computational tools is no longer a specialized skill but a core requirement for translating complex data into actionable public health interventions.

Informatics competencies and computational skills have long been recognized as essential for public health and health services scholars and practitioners (12, 13) and are endorsed by national organizations such as the American Medical Informatics Association (11). Accordingly, public health competency frameworks increasingly incorporate data analytics as a core skill set, emphasizing proficiency in statistical analysis, data visualization, and computational thinking (12, 13). Despite this recognition, a substantial gap remains between educational offerings and workforce needs, as many programs have yet to fully integrate these competencies into their core curricula (14, 15). The rapid emergence of new tools further complicates curriculum development, as traditional academic structures often struggle to adapt quickly. Bridging this gap requires innovative educational approaches that provide accessible, practical training in digital tools and statistical methods, enabling a broader range of learners to participate effectively in a data-driven field (14, 15). Historically, public health informatics training has relied on location-dependent modes characterized by in-person instruction and centralized, institution-based computing resources. While effective in many contexts, this approach introduces structural limitations that can exacerbate educational inequities, particularly due to the prohibitive costs of maintaining advanced hardware and the rigid scheduling of synchronous instruction (16). These barriers disproportionately affect students in low-resourced settings, who may face constraints related to geographic access, institutional infrastructure, and competing personal or professional responsibilities. As a result, traditional training models can inadvertently function as gatekeeping mechanisms, limiting participation and reducing diversity within the computational public health workforce.

To address these limitations, emerging pedagogical approaches emphasize microlearning–an instructional strategy that delivers content in short, modular units to enhance accessibility and support incremental skill development (17, 18). By focusing on concise, targeted learning objectives, microlearning allows learners to build technical competencies at their own pace, effectively democratizing access to professional development. This approach is further strengthened by the use of cloud-based computational tools, including R, RStudio, and platforms such as Google Colab, which shift computational burdens from local hardware to scalable, browser-based environments (19). These technologies reduce both financial and technical barriers, enabling students to engage in advanced data analysis and modeling without requiring costly institutional infrastructure.

Despite these advances, many computational training programs in public health continue to rely on classroom instruction delivered in person through lectures, labs, and workshops (20, 21). Such synchronous, location-dependent models pose ongoing challenges in accessibility, costs, scheduling flexibility, and resource availability (14, 22–24). These challenges are particularly pronounced for students at low-resourced institutions, who may have limited exposure to cloud-based research and face additional barriers to participation (22). In response, remote and asynchronous learning platforms have emerged as promising alternatives, offering flexible, scalable opportunities for students to develop computational skills while engaging in interactive, collaborative learning experiences (7, 14, 25).

### Study goal

This study evaluated the feasibility and effectiveness of a pilot remote training program designed to address gaps in digital skills development among public health students, particularly those from low-resourced institutions. The study addressed the following research questions:

**RQ1:** What are students' levels of satisfaction with the flexible, asynchronous delivery format, and which aspects of the remote learning experience do they perceive as most valuable or most challenging?

**RQ2:** To what extent does participation in a remote, asynchronous, cloud-based data analytics training program enhance students' practical competencies in data analysis and scientific communication, as demonstrated by their ability to modify R code, interpret regression results, and produce scientific abstracts using existing public health datasets?

## Materials and methods

### Context

The course, titled “Introduction to Cloud Data Analytics for Health Services Research,” was delivered remotely and asynchronously over a three-week period from November to December 2025. It was designed to introduce students to foundational computational tools and scientific communication skills relevant to health services research. The flexible format allowed students to engage with course materials at their own pace while participating in interactive, collaborative learning activities.

Through a combination of hands-on training, guided assignments, and independent exploration, students developed skills in data analysis and visualization using R, RStudio, and Google Colab. These skills were applied in an individual research project using data from the Health Information National Trends Survey 7 (HINTS 7). By the end of the three modules, students were able to run and modify analytical code in R, interpret regression model results, and communicate findings through a scientific abstract.

The course was structured around the following learning objectives: (1) develop technical proficiency in R and Google Colab for data cleaning, analysis, and visualization; (2) apply coding skills to real-world health services research questions using HINTS 7 data; (3) engage in collaborative, peer-supported learning to strengthen research communication; and (4) produce a scientific abstract and a portfolio of applied projects demonstrating skills in data analysis and scholarly writing.

Overall, the course aimed to build foundational proficiency in both local and cloud-based computational tools for conducting analyses relevant to health services research. It was also intended to support continued learning and prepare students for more advanced academic or professional work in the field. Upon enrollment into the asynchronous course, students received access to the institutional Canvas course that housed three modules with training materials, assignments, and discussion prompts. Students were also instructed to create or use an existing Google account to access the Google CoLab platform. Additional details on module-specific learning objectives, assignments, and discussion activities are provided in the public GitHub Repository (https://github.com/YuliaLevites/2025_cloud_computing). The Google Colab notebooks and R code used in the course are available upon request.

### Participant recruitment

Participants were recruited through targeted outreach to academic advisors and institutional partners within the twenty-two institutions of the Research Centers in Minority Institutions (RCMI) network. This approach was intended to engage students from institutions committed to supporting the training and advancement of researchers from historically underrepresented groups in biomedical and health sciences.

Academic advisors at participating RCMI institutions were contacted directly with a course overview, eligibility criteria, and enrollment instructions, and were asked to share this information with eligible undergraduate and graduate students. This strategy facilitated broad dissemination across multiple campuses while targeting students with interests in health services research, data analytics, and scientific communication. It also aimed to reach learners with limited prior exposure to cloud-based analytical tools, thereby supporting capacity-building and research skill development across the RCMI community.

### Data collection and measures

Data were collected through pre- and post-program surveys administered via the Canvas Learning Management System (LMS). The pre-program survey captured demographic characteristics, including age, gender identity, race, ethnicity, institutional affiliation, academic level, field of study, geographic background, and parental education.

The survey also included five multiple-choice questions assessing baseline knowledge of cloud computing, the use of R in Google Colab, the structure of a scientific abstract, the interpretation of logistic regression coefficients, and modifications to R code for analyzing HINTS 7 data. The post-program survey included the same five questions to assess changes in knowledge following program participation.

Self-rated competencies were assessed retrospectively in the post-program survey using a retrospective pre–post design. Participants rated their perceived abilities both before and after the program at a single time point using a 5-point scale (1 = no ability to 5 = great ability). Nine competencies aligned with the course objectives were assessed: (1) understanding the purpose and applications of R in data analysis; (2) installing R and RStudio; (3) navigating the RStudio interface and executing basic commands; (4) using Google Colab to open, run, and save R notebooks; (5) running and modifying R code for public health data analysis; (6) interpreting regression coefficients and odds ratios; (7) explaining the purpose of scientific communication; (8) describing the structure of a scientific abstract; and (9) producing a clear and well-written abstract.

The students' ability to produce a scientific abstract reporting on the analyses they learned and completed during the training was used as an observable, behavioral outcome. In addition, students were to capture and upload screen captures that were used to verify that they completed the assignments correctly.

Student satisfaction was measured using Likert-scale items assessing overall course experience, topic relevance, opportunities for virtual peer interaction, course length, and perceived value for academic development (1 = dissatisfied to 5 = highly satisfied). Perceived difficulty of using Google Colab and learning cloud computing was assessed on a 7-point scale (1 = very difficult to 7 = very easy). Students were also invited to provide open-ended feedback on course duration, materials, office hours, and their overall learning experience.

### Analysis

The main analysis waslimited to students who completed both the pre- and post-program surveys. Descriptive statistics were calculated for demographic characteristics. A retrospective pre–post design was used to assess changes in objective knowledge (26). A composite knowledge score (range: 0–5) was created by summing correct responses across the five knowledge questions, with one point awarded for each correct answer. Paired *t*-tests were used to evaluate differences in mean knowledge scores between the pre- and post-program assessments, with Wilcoxon signed-rank tests conducted as nonparametric sensitivity analyses. For self-rated competencies, median scores were calculated for pre- and post-program ratings, and differences were assessed using Wilcoxon signed-rank tests due to the ordinal nature of the data. Means and standard deviations were calculated for student satisfaction and perceived difficulty measures. To assess potential nonresponse bias, a supplementary analysis compared demographic characteristics and baseline knowledge scores between students who completed both the pre- and post-program surveys and those who completed only the pre-program survey. Fisher's exact tests were used for categorical variables, and the Wilcoxon rank-sum test was used for baseline knowledge score comparisons. All analyses were conducted using R version 4.4.1 and RStudio version 2026.01.0 (27, 28).

### Ethical review

The study was reviewed by the University of Florida Institutional Review Board (IRB) and determined to be exempt (#ET00049833). No compensation was provided to participants, and no individually identifiable information is reported in this manuscript or accompanying materials.

## Results

### **Participant** characteristics

A total of 64 students enrolled in the program, of whom 34 completed the pre-program survey and provided demographic information. Completion of both the pre- and post-program knowledge assessments was required; 19 students met this requirement and were included in the sample for the outcome analysis.

Among participants included in the analysis, most were undergraduate students (89.5%), aged 18–24 years (89.5%), and identified as female (73.7%). Students represented a range of academic disciplines, with the largest proportion from computer science and engineering-related fields. Detailed demographic characteristics are presented in Table 1.

**Table 1:** Demographic characteristics of participants in the Introduction to Cloud Data Analytics for Health Services Research course.

Characteristic	*n*	Percent (%)^*^
*Age*		
18–24	17	89.5
25–34	1	5.3
Prefer not to say	1	5.3
*Gender*		
Female	14	73.7
Male	5	26.3
*Race*		
Asian	6	31.6
Black or African American	5	26.3
White	5	26.3
Multiracial	2	10.5
Prefer not to say	1	5.3
*Ethnicity*		
Hispanic, Latinx, or Spanish Origin	3	15.8
Not Hispanic, Latinx, or Spanish Origin	16	84.2
*Highest level of parents' education*		
High school (including GED)	8	42.1
Associate's degree	1	5.3
Bachelor's degree	3	15.8
Master's degree	6	31.6
Trade school or technical certification	1	5.3
*Academic position*		
Undergraduate Student	17	89.5
Graduate Student	1	5.3
Research Staff	1	5.3
Degree major/discipline^**^		
Computer science/engineering	9	47.4
Health sciences/public health/health professions	7	36.8
Social sciences/business	3	15.8

^*^Percentages may not sum to 100 due to rounding.

^**^Degree majors were grouped into broader categories for presentation.

### Student satisfaction and perceived difficulty

Students reported generally high levels of satisfaction across all assessed domains. On a 1–5 scale, mean ratings ranged from 3.5 to 4.5. Specifically, mean scores were as follows: overall satisfaction with the program, *M*= 4.4 (SD = 0.8), topics addressed, *M*= 4.4 (SD = 0.9), opportunities to interact with peers, *M*= 3.5 (SD = 1.1), length of the program, *M*= 4.2 (SD = 0.9), and value for academic development, *M*= 4.5 (SD = 0.8).

The highest level of satisfaction was observed for value for academic development, with 58% of respondents rating this domain as 5 (“extremely satisfied”). In contrast, satisfaction with opportunities for peer interaction showed greater variability, with only 28% of students selecting the highest rating for this domain compared with the others.

Students also rated the perceived difficulty of key course components on a 1–7 scale. The rating for using the Google Colab notebook was *M*= 5.6 (SD = 1.1), indicating relatively low perceived difficulty. In comparison, learning cloud computing concepts using Google Colab was rated somewhat lower *M*= 4.2 (SD = 0.9), suggesting moderate perceived difficulty.

### Assignment completion

Assignment completion was high overall, with 18 of 19 students (94.7%) completing all three course assignments, posting module-related discussions, and submitting screen captures and abstracts as a culminating assignment and observable assessment of their skill gain.

### Objective knowledge

The mean objective knowledge score increased from *M*= 3.58 (SD = 1.17) on the pre-program assessment to *M*= 4.37 (SD = 1.16) post- program. This improvement was statistically significant (mean difference = 0.79, 95% CI: 0.04–1.54; *t* (18) = 2.22, *p* = 0.039). Findings were consistent in a nonparametric sensitivity analysis using the Wilcoxon signed-rank test (*p* = 0.047).

### Self-rated competencies

Analysis of retrospective pre-post ratings collected in the post-program survey indicated statistically significant improvements in 8 of the 9 assessed competencies (Wilcoxon signed-rank tests, *p* < .05). Median changes calculated as the median of individual post–pre differences ranged from 0 to 2 points. Because the Wilcoxon signed-rank test evaluates paired differences at the individual level, it may yield statistically significant results even when the group-level medians and ranges remain unchanged. No statistically significant improvement was observed for the competency related to producing a clear and readable scientific abstract. Median pre- and post-program ratings (with ranges), along with corresponding *p*-values for changes. are presented in Table 2.

**Table 2:** Self–rated competencies before and after the program (1–5 scale) in the introduction to cloud data analytics for health services research course.

Competency	Pre median (Range)	Post median (Range)	Median change (Post – pre)^*^	*p* value
Understand the purpose and applications of R in data analysis	3 (1–5)	4 (3–5)	1	0.002
Install R and R Studio on your computer	2 (1–5)	5 (3–5)	2	0.002
Navigate the R Studio interface and execute basic R commands and scripts	2 (1–5)	4 (2–5)	1	0.002
Use Google Colab to open, run, and save R notebooks	2 (1–5)	4 (3–5)	1	0.003
Run and modify R code to conduct analyses of a public health dataset	2 (1–5)	4 (3–5)	1	0.003
Interpret coefficients and odds ratios from regression model results and summarize key findings	3 (1–5)	4 (3–5)	1	0.009
Explain the purpose and importance of scientific communication	4 (2–5)	4 (2–5)	0	0.045
Describe the main sections of a scientific abstract and the key questions that an abstract should answer	4 (2–5)	4 (2–5)	0	0.025
Produce a clear and readable scientific abstract	4 (1–5)	4 (1–5)	0	0.078

^*^Ratings were collected retrospectively in the post–program survey using the retrospective survey design.

### Qualitative feedback

Open-ended responses were generally positive. Students highlighted the value of integrating theoretical concepts with hands-on exercises, noting that this approach strengthened their understanding of cloud-based data analytics. One participant emphasized that the course provided practical experience in abstract writing, which helped refine their scientific communication skills. Several students also indicated intentions to apply the skills learned to future academic or research projects.

Suggestions for improvement were varied but constructive. Some students described the course as well-paced and manageable, while one noted it felt slightly too short. Others reported that some materials were somewhat overwhelming, although several participants characterized the course as accessible for beginners. Additional feedback included appreciation for discussion posts as a space to ask questions and a desire for more practice in R coding on further review of key functions introduced during the course.

### Couse completion

A supplementary analysis compared demographic characteristics and baseline knowledge scores between students who completed both the pre- and post-program surveys (*n* = 19) and those who completed only the pre-program survey (*n* = 15). No statistically significant differences were observed between the groups with respect to demographic characteristics or baseline knowledge scores (Supplementary Table 1). Mean ± SD baseline knowledge scores were 3.58 ± 1.17 among students who completed both surveys and 3.60 ± 0.99 among students who completed only the pre-program survey (Wilcoxon rank-sum *p* = 0.971).

## Discussion

### Principal findings

This pilot program demonstrates the effectiveness of a short-form, online microlearning approach for building computational skills among students from diverse disciplinary backgrounds. Participants reported high levels of satisfaction, and both self-rated competencies and objective knowledge scores improved significantly following program completion. Notably, students reported increased confidence in key data analysis skills, including navigating the RStudio interface, executing basic R commands, modifying code for public health datasets, and using Google Colab. These findings indicate that the program effectively supported both practical, hands-on skill development and foundational conceptual understanding.

The three-week, asynchronous format appeared well-suited for students who may face barriers to traditional training, such as scheduling constraints or limited access to computational infrastructure. Significant improvements in objective knowledge and in eight of nine self-rated competencies support the use of brief, targeted training to build foundational skills. These findings also reflect a broader gap between traditional public health curricula and the technical demands of a data-driven workforce. By leveraging accessible, cloud-based tools such as Google Colab, this program offers a scalable model for modernizing computational training in public health.

### Comparison with prior work

The shift toward short-form, asynchronous microlearning represents a critical pedagogical evolution necessary to keep pace with the digital transformation of public health (10). While traditional informatics training has historically relied on location-dependent, in-person instruction, these models often create gatekeeping mechanisms due to the high costs of maintaining institutional hardware and the rigidity of synchronous scheduling. By using a modular, asynchronous format, this program showed how these challenges can be addressed, demonstrating that foundational technical competencies can be developed without the prohibitive resource requirements of centralized, institution-based computing.

Furthermore, this study addresses documented barriers to computational training by providing a scalable alternative to classroom-bound models that often exacerbate educational inequities (9). Our findings confirm that cloud-based platforms like Google Colab effectively shift the computational burden away from local hardware, allowing students in low-resourced settings to engage in advanced data modeling using only a browser. By intentionally recruiting through the RCMI network, this program demonstrates a viable pathway for “democratizing” digital skills—specifically R and cloud analytics—among historically underrepresented groups who may otherwise lack exposure to these essential workforce competencies.

### Limitations

This study has several limitations. First, the small analytic sample size limits statistical power and generalizability. Incomplete post-program survey follow-up introduces the potential for nonresponse bias. However, supplementary analyses showed no significant differences in demographic characteristics and baseline knowledge scores between students who completed both the pre- and post-program surveys and those who completed only the pre-program survey. Second, participants were recruited from 22 institutions within the RCMI network, which may limit the applicability of findings to other populations. Participants may also have had a higher level of baseline interest or motivation in public health data analysis and informatics than the broader student population, introducing the possibility of self-selection bias into the program. Third, the use of a retrospective pre–post design for self-rated competencies may introduce response bias, although this approach helps mitigate response shift bias in self-assessment. Although assignment completion provided an additional objective measure of participant engagement with the program, the study did not include a formal rubric-based evaluation of assignment quality, coding proficiency, or participants' accuracy in performing data analysis tasks. Fourth, the intervention duration was relatively short (3 weeks), and the curriculum focused specifically on introductory R programming and Google Colab-based cloud computing. Therefore, findings may not generalize to longer-term training programs, other computational platforms, or more advanced data science competencies. Additionally, outcomes were measured immediately following the program, and longer-term retention of skills was not assessed. Finally, the absence of a control group precludes causal inference regarding program effectiveness.

Despite these limitations, this study contributes to a growing body of evidence supporting innovative, accessible approaches to computational training in public health. The findings suggest that short, remote, and asynchronous programs can effectively build foundational skills while reducing barriers related to cost, infrastructure, and scheduling. These approaches may be particularly valuable for students in low-resourced settings and those with limited prior exposure to data analytics.

### Implications for practice

These findings reinforce the growing recognition that computational proficiency is a core competency for modern public health professionals (7, 29). By aligning course content with established informatics competency frameworks, including those supported by the American Medical Informatics Association (AMIA), this program translates abstract competency standards into applied, accessible learning experiences. The modular structure—focused on programming fundamentals, cloud-based analysis, and scientific communication—provides a practical template for integrating technical training into existing curricula without requiring extensive restructuring. This approach supports more flexible, skills-based learning pathways that can adapt to rapidly evolving technological demands.

It is important to note that in this program participants worked with deidentified publicly available HINTS 7 data, which did not contain protected health information. However, implementing cloud-based computational training in broader public health education settings may involve additional ethical, governance, privacy, and data security considerations related to handling sensitive health data. Future programs should be mindful of the additional complexities associated with analyzing sensitive health or patient data and consider incorporating training on secure cloud computing practices and data governance requirements as appropriate.

The results also highlight important distinctions in how different types of skills are acquired. While students demonstrated significant gains in technical competencies, no statistically significant improvement was observed in scientific abstract writing. This suggests that higher-order skills, such as scientific communication, may require more sustained practice and iterative feedback than can be provided in a short, asynchronous format. In contrast, procedural and tool-based skills may be more readily developed through modular, self-paced instruction because these competencies involve discrete operational tasks that can be practice incrementally. Prior educational research has suggested that microlearning approaches may be particularly effective for targeted procedural skills acquisition through concise, focused learning activities, while development of higher-order thinking and communication skills may require more interactive and constructivist learning processes involving feedback, reflection, and active knowledge construction (30, 31). Future program designs may benefit from incorporating more structured feedback mechanisms, peer review opportunities, collaborative writing exercises, or extended opportunities for mentored writing practice to better support scientific communication outcomes within asynchronous or microlearning environments.

Finally, the high levels of student satisfaction observed in this study support the acceptability of remote, asynchronous training models. Participants valued the flexibility and accessibility of the format, indicating that rigorous skill development can occur outside traditional classroom settings. These findings challenge the continued reliance on in-person, synchronous instruction and suggest that well-designed online programs can provide an effective and inclusive alternative.

Future studies should incorporate more objective measures of participant performance, such as rubric-based evaluation of assignment quality, coding proficiency, and accuracy in completing data analysis tasks, as well as longitudinal assessment for evaluation of long-term skill retention.

## Data Availability

The original contributions presented in the study are included in the article/Supplementary material, further inquiries can be directed to the corresponding author.
