# Supplementary material for: Democratizing computational skills: evaluating an asynchronous microlearning framework for cloud-based data analytics in health services research
Source: Front Public Health. 2026 Jun 16;14:1868973. doi: 10.3389/fpubh.2026.1868973 (PMC13314753; doi:10.3389/fpubh.2026.1868973)
Supplement: Supplementary file 1 [file Table_1.docx]

Supplementary Table 1. Comparison of Baseline Characteristics Between Students Who Completed Versus Did Not Complete the Post-Program Survey Among Pre-Program Survey Respondents

| **Characteristic** | **Completed post-survey (n=19)** | **Did not complete post-survey (n=15)** | **p-value** |
| --- | --- | --- | --- |
| **Age** |  |  | 0.428 |
| 18-24 | 17 (89.5) | 12 (80.0) |  |
| 25-34 | 1 (5.3) | 1 (6.7) |  |
| 45-54 | 0 (0.0) | 2 (13.3) |  |
| Prefer not to say | 1 (5.3) | 0 (0.0) |  |
| **Gender** |  |  | 0.675 |
| Female | 14 (73.7) | 11 (73.3) |  |
| Male | 5 (26.3) | 3 (20.0) |  |
| Non-binary / third gender | 0 (0.0) | 1 (6.7) |  |
| **Race** |  |  | 0.339 |
| Asian | 6 (31.6) | 2 (13.3) |  |
| Black or African American | 5 (26.3) | 8 (53.3) |  |
| Multiracial | 2 (10.5) | 3 (20.0) |  |
| Prefer not to say | 1 (5.3) | 0 (0.0) |  |
| White | 5 (26.3) | 2 (13.3) |  |
| **Ethnicity** |  |  | 1.000 |
| Hispanic, Latinx, or Spanish Origin | 3 (15.8) | 2 (13.3) |  |
| Not Hispanic, Latinx, or Spanish Origin | 16 (84.2) | 13 (86.7) |  |
| **Academic position** |  |  | 0.313 |
| Faculty | 0 (0.0) | 2 (13.3) |  |
| Graduate Student | 1 (5.3) | 2 (13.3) |  |
| Research Staff | 1 (5.3) | 1 (6.7) |  |
| Undergraduate Student | 17 (89.5) | 10 (66.7) |  |
| **Degree major / discipline** |  |  | 1.000 |
| Computer science / engineering | 9 (47.4) | 7 (46.7) |  |
| Health sciences / public health / health professions | 7 (36.8) | 6 (40.0) |  |
| Social sciences / business | 3 (15.8) | 2 (13.3) |  |
| **Highest level of parents' education** |  |  | 0.220 |
| Associate's degree | 1 (5.3) | 2 (13.3) |  |
| Bachelor's degree | 3 (15.8) | 2 (13.3) |  |
| High school (including GED) | 8 (42.1) | 6 (40.0) |  |
| Master's degree | 6 (31.6) | 1 (6.7) |  |
| PhD, MD, JD, PharmD, or other terminal degree | 0 (0.0) | 3 (20.0) |  |
| Trade school or technical certification | 1 (5.3) | 1 (6.7) |  |
| **Baseline knowledge score (Mean ± SD)** | 3.58 ± 1.17 | 3.6 ± 0.99 | 0.971 |

Percentages are column percentages and may not sum to 100 due to rounding.

Fisher’s exact tests were used for categorical variables. Baseline knowledge scores were compared using the Wilcoxon rank-sum test.
